# Fat enhanced leukocyte-platelet-rich fibrin versus fascia lata in endoscopic reconstruction of CSF leaks

**DOI:** 10.1007/s00405-023-08010-z

**Published:** 2023-05-16

**Authors:** Ahmed Aly Ibrahim, Ahmed Yoneis, Ahmed Elsakka, Samy Elwany

**Affiliations:** 1 Department of Otolaryngology, Alexandria Faculty of Medicine, Alexandria University, Alexandria, Egypt; 2 Egyptian Foundation for Metabolic Researches, Alexandria, Egypt

**Keywords:** L-PRF, Leukocyte-platelet-rich fibrin, Tissue engineering, CSF leak, Skull base ·

## Abstract

**Purpose:**

The aim of this study was to use a new biological active fat enhanced leukocyte-platelet-rich fibrin membrane (L-PRF) for skull base defect reconstruction and compare its validity and reliability with the time-honored fascia lata.

**Methods:**

This prospective study was conducted on 48 patients with spontaneous CSF leaks who were divided into 2 matched groups by stratified randomization, 24 patients in each group. In group A we performed multilayer repair using fat enhanced L-PRF membrane. In group B we used fascia lata for the multilayer repair. In both groups we enforced the repair with mucosal grafts/flaps.

**Results:**

The two groups were statistically matched for age, sex, intracranial pressure, and site and size of the skull base defect. There was no statistically significant difference between the two groups regarding the outcome of the repair or recurrence of CSF leak during the first postoperative year. Meningitis occurred in one patient in group B and was successfully treated. Another patient in group B developed thigh hematoma which resolved spontaneously.

**Conclusion:**

The fat enhanced L-PRF membrane is a valid reliable option in repair of CSF leaks. The membrane is autologous, readily available, easily prepared, and has the advange of including stromal fat, stromal vascular fraction (SVF), and leukocyte-platelet-rich fibrin (L-PRF). The present study showed that fat enhanced L-PRF membrane is stable, non-absorbable, not liable to shrink or become necrotic, and can establish good seal of the skull base defect and further enhance the healing process. The use of the membrane also has the advantage of avoiding thigh incision and possible hematoma formation.

## Introduction

During the last two decades, endoscopic endonasal repair of skull base (SB) defects has been markedly advanced. The evolution in endoscopic instrumentations, endoscopic imaging technology, and navigation systems allowed tremendous expansion in endoscopic resection of skull base tumors resulting in larger SB defects that pose surgical challenges for repair. This expansion necessitated comparable progress in reconstruction techniques.

The key factors that contributes to SB reconstruction failure are the materials used for reconstruction, size and site of the defect, the presence and flow rate of intraoperative CSF leak, and previous or future radiation therapy [1]. Several reconstructive materials have been reported in the literature including biomaterials such as Gelfoam and polymethylmethacrylate, non-vascularized free grafts, and vascularized pedicled flaps [2].

Vascularized pedicled flaps, especially nasoseptal flap, have superior outcomes to free grafts because the healing is accelerated by restoring the local blood [3] and significantly decrease the rate of CSF leak from 16 to 5% [4, 5], however, the availability of these flaps may be compromised by several factors such as invasion of the flap site by the disease and injury of the blood supply during surgery [6]. Additionally, donor site complications including excessive crusting, septal perforation, mucocele formation, flap necrosis, and synechia should be considered [7].

Leukocyte-platelet rich fibrin (L-PRF) is a second-generation biodegradable autologous blood preparation. It is widely used in different surgical fields due to its ability to accelerate tissue healing by neovascularization, tissue remodeling, anti-infectious activities, and being a good sealant with marvelous adhesive character. [8] L-PRF enriches the surgical fields with leukocyte, growth factors such as vascular endothelial growth factor (VEGF), Platelet-derived growth factor (PDGF),) matrix glycoproteins and cytokines (interleukin-1b and tumor necrosis factor α) [9]. This encourages wound healing by angiogenesis, re-epithelization, extracellular matrix formation, and migration of stem cells differentiating into osteoblasts [10]. So, it was postulated that platelet rich fibrin (PRF) enhances and accelerates the healing of bone defects [10, 11].

Tissue engineering of autologous adipose tissue has overcome major drawbacks of fat as a reconstructive material such as tissue shrinkage and high susceptibility to ischemia with necrosis and apoptosis of the implanted adipocytes [12]. Stromal vascular fraction (SVF) that can be easily extracted from adipose tissues, contains multipotent adipose-tissue-derived stem cells (ASCs) that can differentiate into cells of mesodermal origin in vitro, e.g., osteoblasts, chondrocytes, and adipocytes [13, 14]. This capability gives SVF an ideal and promising property when used adjuvant to L-PRF and stromal fat as a reconstructive material for osteodural defects [15].

The enhancement of the fat graft by blood derived products such as L-PRF has encouraged the graft survival by different mechanisms [16]. Vascularization of the graft is augmented by proangiogenic factors secreted by L-PRF which decreases the risk of ischemia. Besides, the anti-inflammatory cytokines released by L-PRF guard against graft degeneration. Additionally, maturation of preadipocytes is improved by several PRF-secreted factors. In this study, we assess the reliability of using fat enhanced L-PRF membrane as an active autologous material for endoscopic reconstruction of spontaneous CSF leaks.

Although endoscopic CSF leak repair for spontaneous CSF leaks has a high rate of success without specific consideration towards the grafting material, fascia lata is needed in some cases. The aim of this study was to use a new biological active fat enhanced leukocyte-platelet-rich fibrin membrane (L-PRF) as a less invasive alternative to facia lata for skull base defect reconstruction and compare the validity and reliability of both techniques.

## Materials and methods

This prospective controlled clinical study was conducted on 48 patients with spontaneous CSF rhinorrhea admitted to our University Hospital from Janurary 2017 till December 2021.

The study was conducted after approval of the Ethical Committee of the Faculty of Medicine. Informed consents was obtained from all patients according to the Helsinki declaration.

Exclusion criteria:

1. Size of the defect > 3 cm
2. Patients having any coagulopathy or receiving anticoagulation therapy.
3. Patients with nasal polyposis or sinusitis.

Patients included in the study were random;y divided into two matched groups (stratified randomization):

- *Group A*: (24 patients): Multilayer repair of CSF leak was done using fat enhanced L-PRF membrane which was processed by clinical pathologist.
- *Group B*: (24 patients): Fascia lata was used instead of the L-PRF membrane for the multilayer repair.

In both groups local nasal flaps, and free mucosal graft were used to enforce the repair. Fat plugs were also used when needed.

### Preparation of the autologous L-PRF graft

#### Preparation of L-PRF and serum remnants of thrombin

**Figure1:**
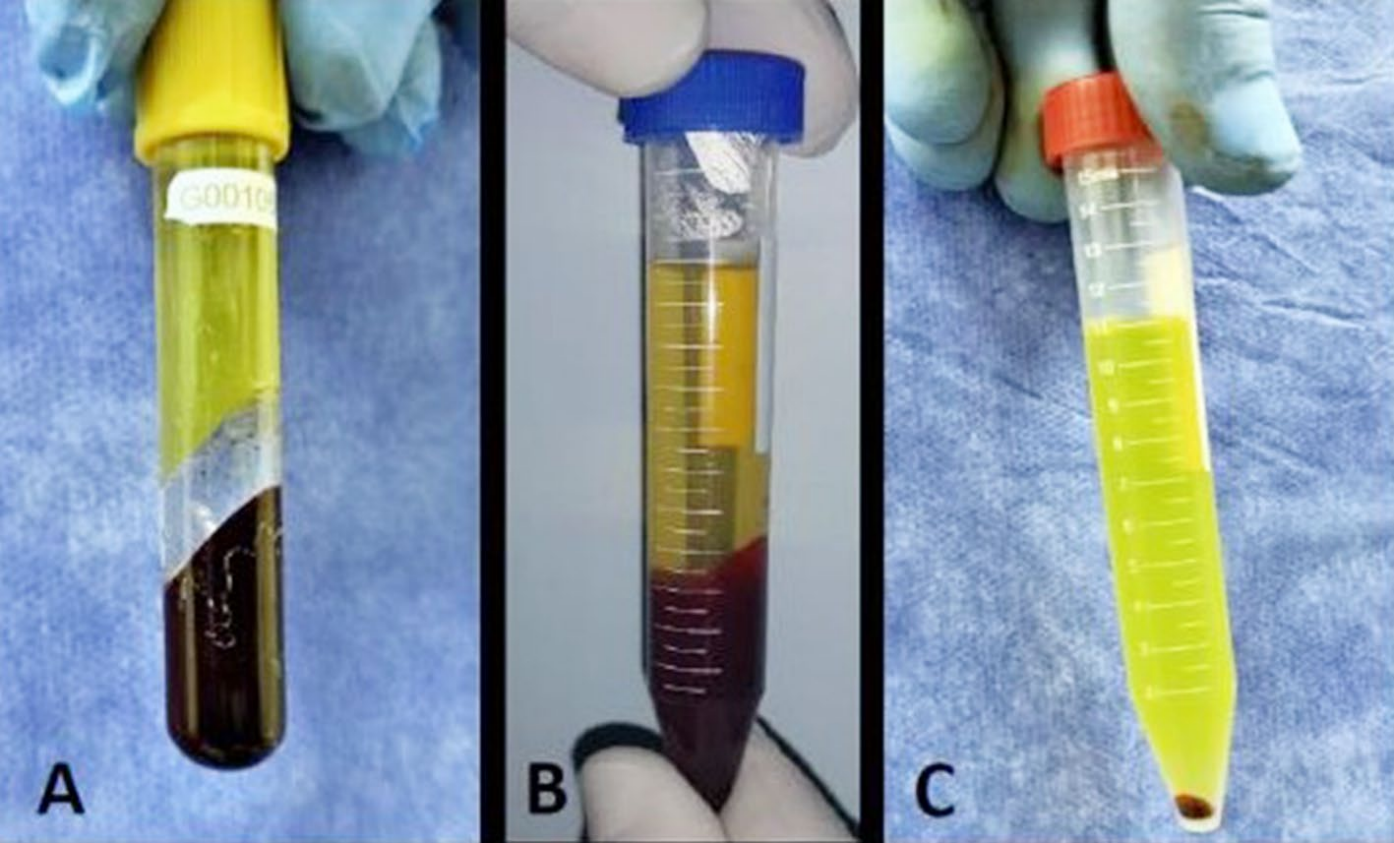
Preparation of L-PRF and serum remnants of thrombin. **A** Thrombin remnants **B** citrated blood after centrifugation showed lower of RBCs, middle layer of platelets and leukocytes, and upper layer plasma containing fibrin. **C** Centrifugated leukocytic platelet “red pellets at the bottom” and plasma containing fibrin “yellow part”

Sixty mL of whole venous blood was obtained from the patient without anticoagulants. Twenty ml of this blood was placed in a sterile vacutainer gel containing tubes with clot activator without any anticoagulant. The tubes were left at room temperature for 10 min for complete clot formation and then were centrifuged for 10 min at 3000 rpm for separation of serum with thrombin remnants (Fig. 1a). The remaining 40 mL was placed in four sterile falcon tubes with 15% sodium citrate as anticoagulant and centrifuged immediately for 15 min at 3200 rpm. After centrifugation, plasma with platelet and puffy coat layer (fibrin part) were transferred to sterile falcon tubes (Fig. 1b, c).

#### Stromal fat harvesting

**Fig. 2:**
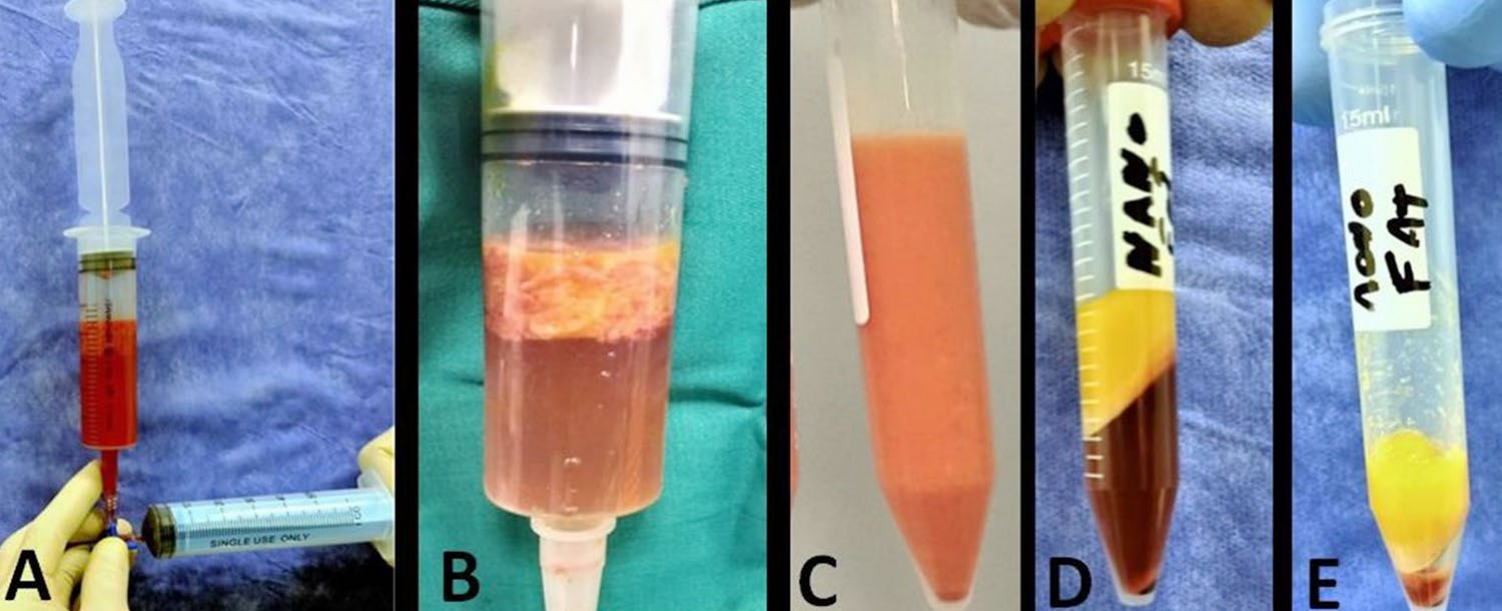
Fat processing. **A** Several washing **B** mechanical digestion **C** fat after washing and mechanical digestion. **D** Fat after centrifugation **E** stromal fat cells with SVF

After induction of anesthesia, 10 mL of fat was aspirated from the abdominal wall using a liposuction cannula (blunted end syringe gauge 14) over 20 min. The aspirated fat underwent several processes of washing by physiologic saline to discard RBCs, then was put inside a special grinder for mechanical digestion of adipocytes, then was placed into two separate sterile falcon tubes (Fig. 2a–c). Each tube was centrifuged at 3000 rpm for 10 min after which the fat was separated into 3 layers: Oil (upper layer), Stromal fat cells (middle layer), and Liquid layer containing substantial vascular fraction (SVF) (lower layer) (Fig. 2d, e).

#### Preparation of fat enhanced L-PRF membrane

**Fig. 3:**
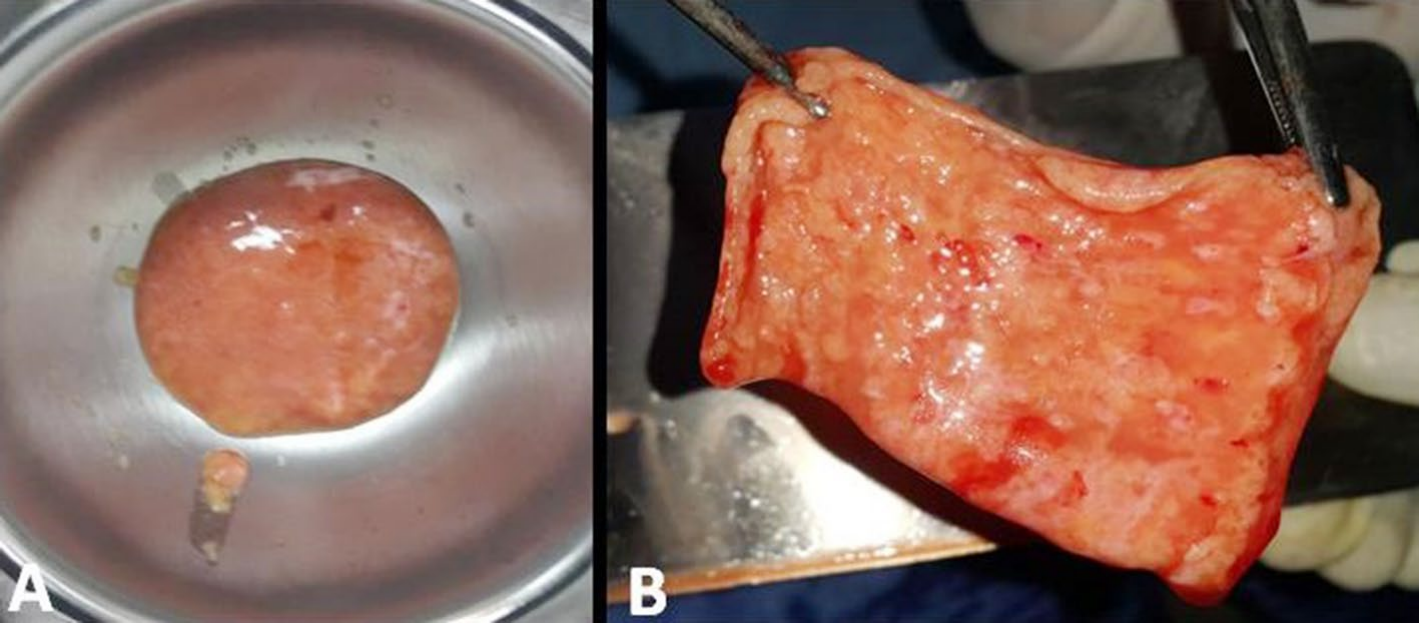
Final steps in preparation of the L-PRF membrane. **A** mixing the fat, thrombin, and fibrin **B** the fat enhanced L-PRF membrane

The stromal fat and SVF mixed with fibrin with a ratio of 1:10 respectively. Thrombin containing calcium chloride was added to the mixer by ratio 1:2 respectively. The final mixture (fat, fibrin, and thrombin) spread as a thick layer in a round sterilized container and left at room temperature for 5 min (Fig. 3a). then, a gel-like membrane formed at the base of the container was transferred to a dental PRF compression box and compressed to form a uniform water-impermeable membrane that can be applied as a graft for the boney defect (Fig. 3b).

### Surgical technique

All operations were performed by the same surgical team under general anesthesia using the endoscopic transnasal approach. The skull base defect was identified after fulguration of the meningoencephalocele, if present, using bipolar forceps and the mucosa surrounding the margins of the bony defect was elevated.

**Fig. 4:**
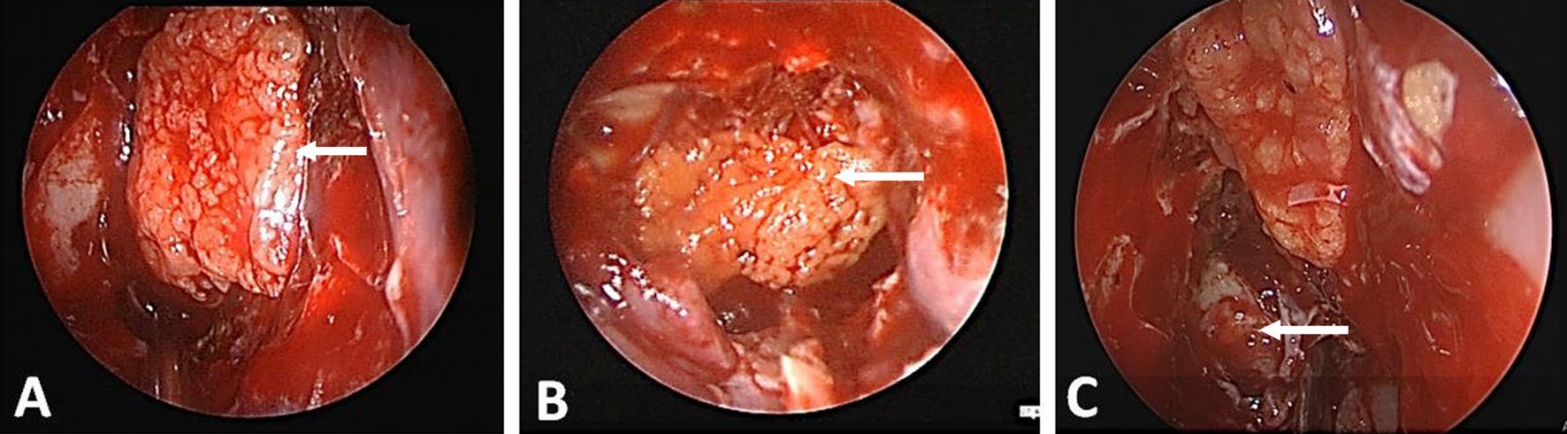
Endoscopic view of fat enhanced L-PRF membrane in different sites. **A** Cribriform defect **B** sphenoid defect **C** posterior frontal table defect

For group A, fat enhanced L-PRF membrane was prepared during the surgery and the membrane was applied as underlay and onlay layers (Fig. 4). The onlay membrane was then covered by mucosal flap/graft. Fat plugs were used whenever needed.

For group B fascia lata was used instead of the L-PRF membrane. Otherwise, the repair was like the repair performed for group A patients.

Reconstructive materials were further supported with Surgicel and/or Gelfoam and a Merocel pack was then inserted. We did not use lumbar drains for any of the cases.

Both groups received the same postoperative protocol. The postoperative follow-up period lasted for one year and included nasal endoscopy every 2 weeks in the first three months then monthly for nine months. The absence of CSF leak within the first year of the repair was considered a successful repair. [17].

### Statistical analysis

Statistical analysis was performed using SAS/STAT 9.22 (SAS Power and Sample Size). The sample size of the study was calculated so that the power of the study would be 80%. The two groups of the study were well matched. The low variability between the two groups decreased random sampling errors and increased the power of the study. Paired student’s *t* test was used to compare means. Chi squared test was used to compare categorical variables A *p* value of less than 0.05 was con- sidered statistically significant.

## Results

Group A consisted of 24 patients, 19 females and 5 males ranging in age from 30 to 52 years old (mean 42.78 years) and the average BMI was 28.1 ± 4.2 kg/m^2^. Group B consisted of 24 patients, 20 females, and 4 males ranging in age from 31–53 years (mean 40.29 years) and the average BMI was 27.8 ± 4.3 kg/m^2^. Table1 shows the demographics of the two groups.

**Table 1:** Demographic data of the two groups

Data	Group A	Group B	*p*
	*N* = 24	*N* = 24	
Age (years)	42.7 (30–52)	40.29 (31–53)	0.6511
Sex			0.7115
Females	19 (79.2%)	20 (83.33%)	
Males	5 (20.8%)	4 (16.7%)	
Body mass index (BMI, Kg/m^2^)	28.1 ± 13%	27.8 ± 4.2	0.5964
Smoking history			0.5825
Smoker	3 (12.5%)	4 (16.7%)	
Non smoker	21 (87.5%)	20 (83.3%)	

Table 2 compares the operative data of the two groups. There was no significant difference in intracranial pressure between the two groups (16.5 cm H_2_a in group A versus 18.8 cm H_2_a in group B). A meningoencephalocele at the defect site was found in 8 (33.3%) group A patients versus 6 (25%) group B patients. The difference between the two groups was statistically insignificant (*p* > 0.05).

**Table 2:** Comparison of the operative data of the two groups

Operative data			*p*
	Group A	Group B	
Intracranial pressure (ICP)	16.5 ± 6.1 cm H_2_0	18.8 ± 5.6 cm H20	0.616
Size of defect			
< 1 cm	15 (62.5%)	14 (58.4%)	0.308
1–2 cm	7 (29.2%)	8 (33.3%)	0.447
2–3 cm	2 (8.3%)	2 (8.3%)	0.435
Site of the defect			
Cribriform plate/ethmoid fovea	13 (54.1%)	15 (62.5%)	0.327
Sphenoid sinus	8 (33.3%)	6 (25.00%)	0.299
Frontal (posterior table)	3 (12.5%)	3 (12.5%)	0.446
Meningocele/encephalocele	8/24 (33.3%)	6/24 (25%)	0.477
Adjunctive grafting materials			0.8612
Mucosal graft	19	20	
Mucosal flap	5	4	
Fat plug	10	8	

The most common leak location in group A was the cribriform plate/ethmoid fovea (54.1%) followed by sphenoid sinus (33.3%), and the posterior table of the frontal sinus (12.5%). The most common leak location in group B was the cribriform plate/ethmoid fovea (62.5%) followed by sphenoid sinus (25%), and the posterior table of the frontal sinus (12.5%). The difference between the two groups was statistically insignificant (*p* > 0.05).

The size of the defect was measured intraoperatively by a cottonoid pledget that was properly cut out to reproduce the defect area [18]. The defect size in group A in the longest dimension was < 1 cm in 14 patients (58.4), 1–2 cm in 7 patients (29.2%), and 2–3 cm in 2 patients (8.3%). The defect size in group B in the longest dimension was < 1 cm in 15 patients (62.5), 1–2 cm in 8 patients (33.3%), and 2–3 cm in 2 patients (8.3%). There were no statistically significant differences between the two groups. Likewise, there was no significant difference between the 2 groups regarding the adjunctive grafting material used during surgery (*p* > 0.05).

Recurrence of CSF leak at the primary site (Table 3) occurred in one patient in each group. In group A, the patient had recurrence three weeks following repair using fat enhanced L-PRF membrane. The defect size was 16 mm in the sphenoid sinus with opening ICP 18 cm H_2_O.

**Table 3:** Comparison between the two studied groups regarding the postoperative outcome and complications

	Group (A), *N* = 24		Group (B), *N* = 24		*p*
	No	%	No	%	
Early CSF leak	1	4.16	**1**	8.33	0.4993
Meningitis	0	0.00	1	4.16	0.3122
Headache	7	29.2	9	37.5	0.5462
Thigh hematoma	0	0.00	1	4.16	0.3122

In group B, the patient experienced recurrence two months after repair using fascia lata and nasoseptal flap. The defect measured 18 mm in the cribriform plate with an opening ICP 20 cm H_2_O.

There was no statistically significant difference between two groups regarding postoperative complications. Postoperative meningitis was diagnosed in one patient in group B. Also, another patient in group developed thigh hematoma at the site of harvesting fascia lata. The hematoma resolved spontaneously.

## Discussion

The main aim of reconstruction of osteodural skull base defects is to create a watertight barrier between the arachnoid space and the sinonasal tract without endangering ocular and neurovascular structures and to avoid major morbidities at the donor site of reconstructing materials [4]. With the advancement in endoscopic approaches, the success rate of reconstruction of small SB defects is greater than 95% [19, 20], while the matter is still challenging on dealing with larger defects.

L-PRF has been widely introduced in different surgical specialties such as neurosurgery, oral and maxillofacial surgery, general surgery [21], orthopedic surgery, ophthalmology, and sports medicine [22] due to its advantages of being inexpensive, autologous, easily prepared, and rich of Fibrin, platelets, growth factors, and leukocytes. These components play a major role in the healing process [23].

In 2016, Soldatova et al. [17] assessed for the first time the potential utility of L-PRF in SB reconstruction. It was found that the healing process had markedly enhanced with improvement of crusting scale score. A recent study documented the role of L-PRF in neoossification when used as adjuvant material in SB reconstruction [24]. Rasmussen et al. [25] reported using the L-PRF membrane as a reconstructive material following the transsphenoidal approach for sellar lesions.

We attempted in our study to use a biological active membrane having the privileges of stromal fat, SVF, and L-PRF to modify the physical character of L-PRF and to provide a permanent stable sealing of the skull base defect. The L-PRF membrane proved to be stable. non-absorbable and is not liable to shrink or become necrotic.

There was no statistically significant difference between the two groups regarding the success of the repair and/or postoperative complications [25]. A single patient in group B developed thigh hematoma, following harvesting a fascia lata graft, that resolved spontaneously. This adds another advantage for using the autologous membrane which avoids incision and hematoma formation at the donor site. Another patient in the same group developed meningitis and was treated successfully.

The limitations of this study are the relatively small sample size of the two groups and the lack of long-term follow-up to detect the delayed CSF leaks. Another issue is that the size of the skull base defects included in the study was less than 3 cm, so the reliability of using fat enhanced L-PRF membrane in larger skull base defects has to be further evaluated in the future. While the current study had no complications from the liposuction, it is likely too early to conclude that there are no donor site complications associated the liposuction necessary for L-PRF.

## Conclusions

We proposed the use of fat enhanced L-PRF membrane as a reliable option for endoscopic repair of SB osteodural defects. The fat enhanced L-PRF membrane is a readily available easy-to-prepare autologous non-absorbable biological active membrane that enhances the healing process and provides stable watertight seal of the skull base defect. Furthermore, it helps in avoiding complications of the donor site. We recommend performing further studies with a larger sample size and larger skull base defectsfor more evaluation of L-PRF skull base repair and its longterm durability.

## Data Availability

The authors confirm that the data supporting the findings of this study are available within the article.
